# Effects of bifrontal-transcranial direct current stimulation combined with music listening on sleep quality, cortical activation and functional connectivity in patients with insomnia: a randomised controlled trial by fNIRS

**DOI:** 10.3389/fpsyt.2026.1763543

**Published:** 2026-04-29

**Authors:** Qin Shi, Siyan Cai, Yingjie Fan, Xuyan Ren, Xiaotong Zu, Tianpei Xie, Chengcheng Zhang, Dejing Cheng, Huifang Tian, Min Su

**Affiliations:** 1 Department of Gynaecology and Obstetrics, The Fourth Affiliated Hospital of Soochow University (Suzhou Dushu Lake Hospital), Suzhou, Jiangsu, China; 2 Institute of Rehabilitation, Soochow University, Suzhou, Jiangsu, China; 3 Department of Rehabilitation Medicine, The Fourth Affiliated Hospital of Soochow University (Suzhou Dushu Lake Hospital), Suzhou, Jiangsu, China

**Keywords:** functional connectivity, functional near-infrared spectroscopy, insomnia, music listening, transcranial direct current stimulation, verbal fluency task

## Abstract

**Background:**

Although music listening and transcranial direct current stimulation (tDCS) alone have certain effects in the treatment of insomnia, the sleep regulatory effects and neural mechanisms of the combined treatment in patients with insomnia disorder (ID) are unclear. This study aimed to investigate the efficacy of combined bifrontal-tDCS (F3: anode, F4: cathode) with music listening in patients with ID using functional near-infrared spectroscopy (fNIRS).

**Methods:**

76 ID patients were randomly divided into an intervention group (n=38) and a control group (n=38), and received 4 weeks of a total of 20 sessions of music + tDCS therapy and music + sham tDCS therapy (30-second stimulation with fade-in/fade-out to mimic somatic sensations), respectively. The Pittsburgh Sleep Quality Index Scale (PSQI), Self-rating Depression Scale (SDS), Self-rating Anxiety Scale (SAS), and Perceived Stress Scale (PSS-14) were compared between the two groups before and after treatment. Oxy-haemoglobin (HbO_2_) concentration and functional connectivity (FC) were assessed during the verbal fluency task using fNIRS.

**Results:**

Compared with the control group, the PSQI total score (mean difference: -2.57 points, 95% CI: -4.43 to -0.71, *p* = 0.001), PSQI sub-scores except “sleep disturbance and daytime dysfunction”, SDS and SAS scores of the intervention group improved significantly after treatment. It was observed by fNIRS that the HbO_2_ concentration in the medial prefrontal cortex (mPFC), left dorsolateral prefrontal cortex (DLPFC), right ventrolateral prefrontal cortex, and right superior frontal cortex (SFC) increased significantly after treatment in the intervention group but was not superior to the control group. In addition, the FC enhancement of left SFC-left DLPFC and left SFC-mPFC after treatment was significantly better in the intervention group than in the control group, and the PSQI improvement was positively correlated with the FC enhancement of channel-averaged and left SFC-right DLPFC.

**Conclusions:**

Combining bifrontal-tDCS with music listening is more helpful in improving sleep quality and prefrontal functional connectivity in ID patients compared with music listening alone. For ID patients, music electrical stimulation headphones may be a safe, effective, and convenient new treatment strategy.

**Clinical trial registration:**

https://www.chictr.org.cn/, identifier ChiCTR2400086233.

## 1 Introduction

In recent years, the prevalence of insomnia disorder (ID) has been increasing year by year and showing a youthful characteristic against the background of the accelerating pace of social life and the significant increase in work pressure (1). ID is one of the most common clinical sleep disorders, which is mainly manifested by difficulty in falling asleep, reduced sleep quality and efficiency, and disturbed nighttime sleep, which in turn leads to daytime fatigue, mood fluctuations, cognitive impairment, and other physiological and Psychological problems (2). Long-term insomnia may lead to decreased immune function and increased risk of cardiovascular and gastrointestinal diseases and complications such as blood glucose and blood pressure abnormalities (3). Although cognitive behavioural therapy is the first-line treatment strategy for ID, it has a long treatment cycle, high requirements for patient compliance, and high heterogeneity of efficacy (4, 5). Pharmacological treatments also have problems such as drug dependence, side effects, and high recurrence rates, which limit their clinical application (6). Accordingly, exploring a safer and more effective insomnia treatment strategy has been the focus of medical research.

Most ID patients may also suffer from depression, anxiety, and/or somatization symptoms. These symptoms are closely and complexly interrelated and often have a reciprocal causal relationship with insomnia (7). Therefore, effectively regulating negative emotions may help break the vicious cycle and improve insomnia. Music listening, as an intervention method that aids sleep by regulating emotions and psychological states, has the potential advantages of being low-cost, easily accessible, and manageable. The mood-regulating effects of music involve complex psychological mechanisms (8), with musical tempo, tonality, and rhythmic structure serving as key elements influencing emotional responses. Research indicates that slow-tempo music can resonate with resting heart rate, inducing physiological relaxation (9). Music predominantly in major keys evokes a bright and soothing sensation, while steady, predictable rhythms can reduce cognitive anxiety and create favourable psychophysiological conditions for falling asleep (10, 11). Based on the above theoretical foundations, interventions using soothing music with these characteristics are expected to enhance relaxation by reducing sympathetic nervous system arousal and diverting attention away from stress, thereby improving sleep quality and alleviating symptoms of anxiety and depression (12, 13).

The non-invasive neuromodulation technique, transcranial direct current stimulation (tDCS), has the advantages of portability, low operating difficulty and safety. By applying weak and continuous low-intensity direct current to specific cortical areas of the brain, tDCS induces changes in cortical activity and neuronal excitability, which in turn promotes neural plasticity (14). The dorsolateral prefrontal cortex (DLPFC) is a key node in the executive control network and plays a crucial role in both sleep and emotional regulation. Patients with ID accompanied by mood disorders often exhibit functional abnormalities in this region, making it a rational target for neuromodulation. Previous studies have reported that bifrontal-tDCS (F3: anode, F4: cathode) targeting the bilateral DLPFC improves subjective and objective sleep quality (15, 16) as well as negative mood (17). From a neurobiological perspective, tDCS primarily modulates cortical excitability (18), while music listening influences emotion and arousal through limbic and autonomic pathways (19). The combination of these two interventions may therefore engage complementary neural circuits, potentially offering greater therapeutic benefits than either approach alone. Given the potential benefits of tDCS for insomnia management and its unique advantages, we considered combining it with music listening in a wearable device and hypothesised that this combined stimulation modality would have a better ameliorative effect on ID patients compared to music listening alone.

Functional near-infrared spectroscopy (fNIRS) is a widely applicable and ecologically valid technique for imaging brain function. Measurement of haemodynamic changes during brain activity based on near-infrared light enables the assessment of dynamic changes in brain function during cognitive tasks in a natural environment (20). The verbal fluency task (VFT) is widely used in neurocognitive assessment, which can effectively detect cortical activation in the frontal and temporal lobes of healthy people and patients with mental health disorders (21), identifying potential biomarkers in psychiatry that can contribute to the diagnosis and treatment of mental disorders (22, 23). Neuroimaging studies have found that ID patients have changes in the structure or function of the prefrontal cortex, manifested as decreased volume, reduced metabolism, and hypoactivity, which may lead to impaired cognitive and executive functions (24). Zhou et al. demonstrated that prefrontal cortical activation was reduced and the average inter-channel functional connectivity (FC) strength was reduced during VFT in patients with chronic ID compared to healthy individuals (25). Patients with short-term ID also exhibited reduced FC between the frontal and temporal cortex, and the FC between the left frontal polar cortex and the right DLPFC was negatively correlated with the Pittsburgh Sleep Quality Index Scale (PSQI) scores (26). These findings suggest that the daytime cognitive impairments observed in patients with ID, such as deficits in executive function and verbal fluency, are closely associated with prefrontal cortex dysfunction. Given that prefrontal function is also critical for sleep regulation via top-down control, the VFT-induced hemodynamic response serves as a sensitive probe for assessing neuroplastic changes related to sleep improvement. Therefore, employing the VFT as the task paradigm can sensitively capture brain functional abnormalities in ID patients and objectively assess intervention-related neuroplasticity.

Functional recovery in most mental disorders and neurological diseases is closely related to cortical functional activation and neural network remodelling (27). However, the neural mechanisms underlying combined tDCS and music listening for insomnia, especially regarding prefrontal function and network connectivity, remain unclear. Therefore, this study aimed to evaluate the efficacy of combined bifrontal-tDCS (F3: anode, F4: cathode) with music listening for ID patients using fNIRS and to elucidate its neural mechanisms to provide a safer, more effective, and more convenient neuromodulation method for insomnia treatment. We hypothesized that adding bifrontal-tDCS to music listening would help to improve sleep quality, reduce negative emotions, and improve prefrontal cortical activation and neural network functional connectivity in ID patients, with efficacy superior to music listening alone.

## 2 Materials and methods

### 2.1 Study design

**Figure 1:**
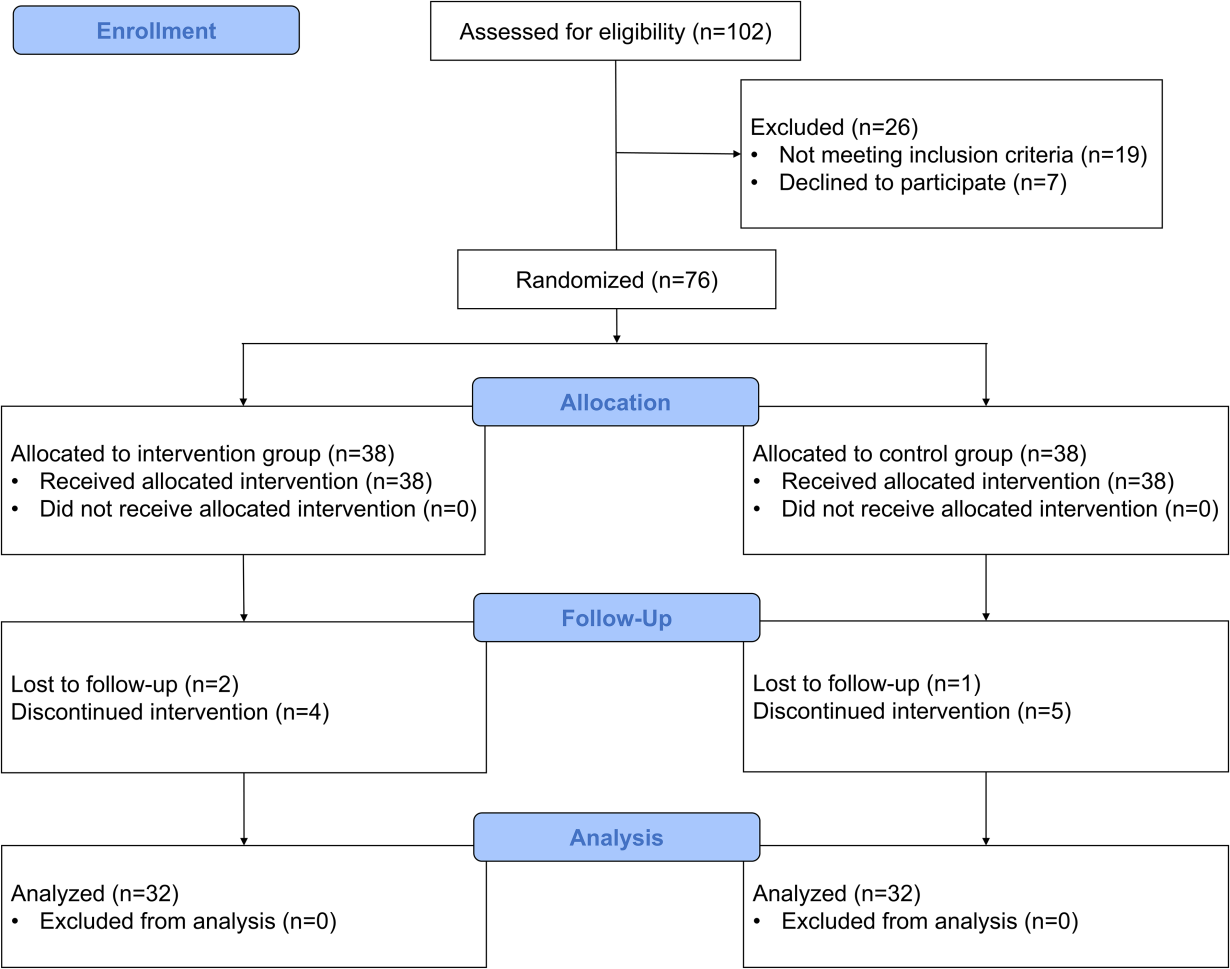
CONSORT flowchart.

This study was a prospective, double-blind, randomised controlled trial. Participants were randomly allocated in a 1:1 ratio to intervention and control groups to receive 4 weeks of music + tDCS therapy and music + sham tDCS therapy, respectively. The trial report followed the CONSORT guidelines (**Figure 1**). The study was reviewed and approved by the Ethics Committee of the Fourth Affiliated Hospital of Soochow University (No: 241024) and registered at https://www.chictr.org.cn/, identifier ChiCTR2400086233) on 27/06/2024. The trial followed the Declaration of Helsinki (2013), and all participants were fully informed about the study procedures and signed an informed consent form.

### 2.2 Participants

The ID patients included in this study were consecutively recruited from July to December 2024 at the Fourth Affiliated Hospital of Soochow University. They were diagnosed by psychiatrists with senior titles and met the diagnostic criteria established by the Diagnostic and Statistical Manual of Mental Disorders, 5th edition (DSM-5) (28). Screening and exclusion were performed in the field using the Chinese version of the Mini-International Neuropsychiatric Interview (MINI) (29).

Inclusion criteria (1): age 18–70 years (2); PSQI score > 5 (30) (3); did not take antipsychotic or sedative-sleeping drugs that may affect the symptoms in the 2 weeks before enrolment (4); right-handed (5); clear consciousness, can cooperate with research. Exclusion criteria: (1) history of drug and alcohol dependence; (2) accompanied by serious physical illness; (3) presence of organic brain lesions or epilepsy; (4) presence of cognitive dysfunction; (5) allergy or intolerance to electrical stimulation; (6) installation of pacemakers or intracranial metallic foreign bodies; (7) long-term daily caffeine intake exceeding 200 mg (equivalent to 2 cups of instant coffee or 3 cups of black tea) and inability to cease consumption for 2 weeks before enrolment and during the trial. Criteria for exclusion and dropout: (1) failure to complete treatment or assessment; (2) poor compliance and withdrawal from the study; (3) exacerbation of the condition or serious adverse reactions during treatment; (4) receipt of any treatment other than the intervention protocol during the study, including the use of rescue sleep medications or any other sleep aids. Compliance was monitored through weekly follow-up interviews.

### 2.3 Sample size

In this study, the PSQI score was used as the main evaluation index. The sample size was calculated using G*Power 3.1.9.4 software. According to the results of the pre-experiment (n=6), the total scores of PSQI in the intervention group and the control group were improved by 5.19 and 2.37 points respectively after 4 weeks of treatment, with a standard deviation of 3.02 points. A two-tailed t-test using a 95% test power and a 0.05 significance level would require a minimum of 31 participants in each group. Considering a 20% dropout rate, a total of 76 participants were required for the trial.

### 2.4 Randomization and blinding

A randomised numerical sequence was generated using SPSS 27.0. Participants were randomly allocated in a 1:1 ratio to intervention and control groups, with 38 cases initially included in each group. Grouping information was placed in opaque envelopes and assigned to participants in sequential order. Researchers responsible for recruitment, allocation of the intervention, and data collection were aware of the grouping. Participants, evaluators, and statistical analysts were unaware of specific groupings. Treatments were administered by trained, licensed physical therapists who gave treatments according to numbered instructions and were not involved in the assessment or analysis of results. To assess the effectiveness of blinding, all participants were asked to guess their group assignment (active stimulation, sham stimulation, or unsure) after the 4-week intervention period.

### 2.5 Intervention

**Figure 2:**
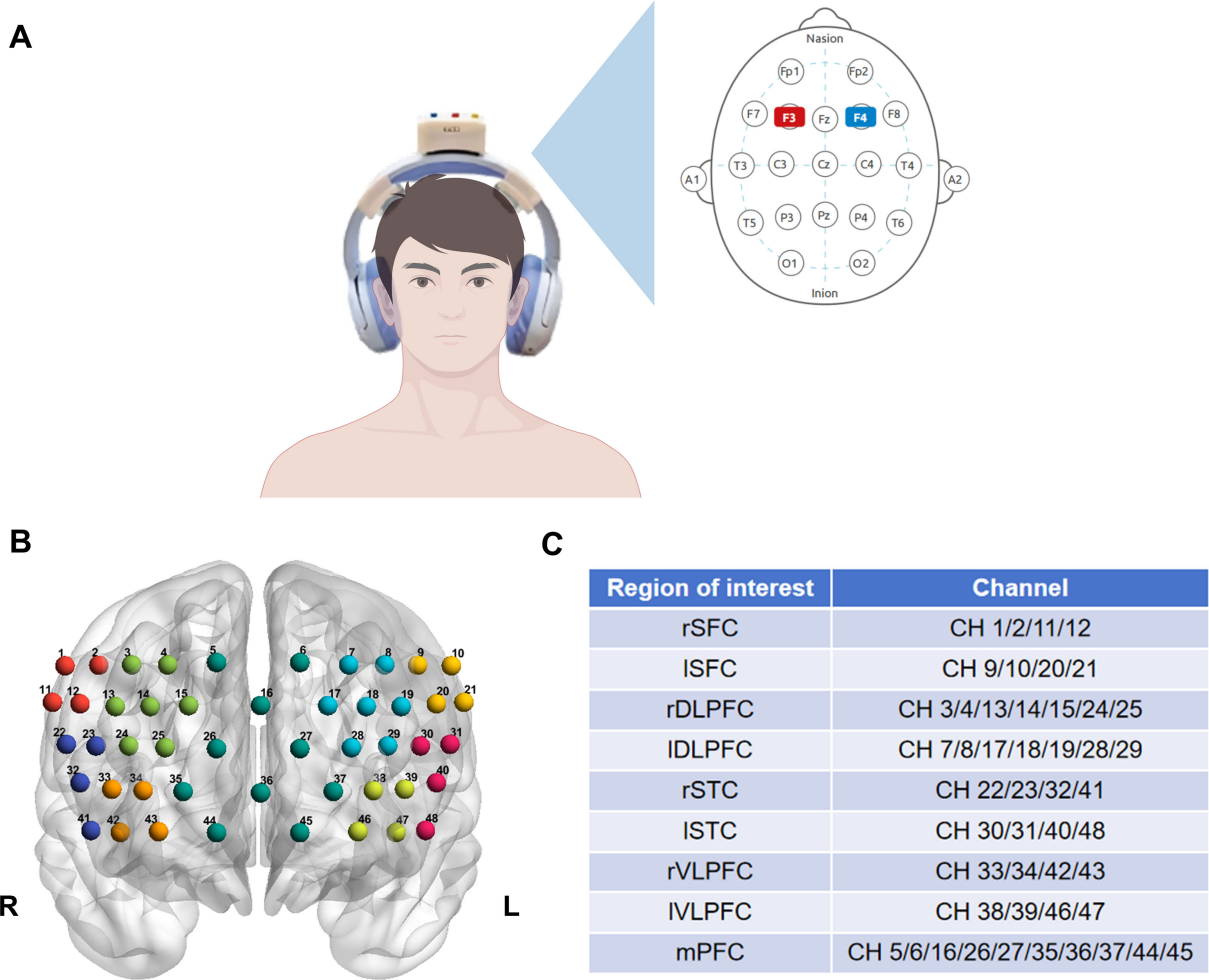
Musical electrical stimulation headphones and distribution of fNIRS channels. **(A)** Schematic diagram of the musical electrical stimulation headphones device and tDCS target brain regions. **(B, C)** Distribution map of fNIRS 48 channels and regions of interest map. SFC, superior frontal cortex; DLPFC, dorsolateral prefrontal cortex; STC, superior temporal cortex; VLPFC, ventrolateral prefrontal cortex; mPFC, medial prefrontal cortex; R, right; L, left; CH, channel. The 3D brain image was visualized with the BrainNet Viewer [(58), http://www.nitrc.org/projects/bnv/].

In this study, we used the musical electrical stimulation headphones model V1 (Suzhou Linghuier Technology Co., Ltd.) to intervene with participants. The design principles and technical details of this device have been reported in detail in a previous study (31). The device consists of two modules: electrical stimulation and musical stimulation (**Figure 2A**).

The electrical stimulation module contains a tDCS stimulator and two gel electrodes (model: EEGT08, contact area: 3 cm × 2 cm). According to the electroencephalogram (EEG) 10–20 system localisation method, the bilateral DLPFC of the brain was taken as the stimulation targets. In which anodic stimulation was applied to the left DLPFC (F3 region) and cathodic stimulation was applied to the right DLPFC (F4 region). After the electrodes were assembled, the device was worn on the head with the indicator light facing forward relative to the patient. Align the anode electrode with the F3 region and the cathode electrode with the F4 region. Tousle the hair as much as possible to ensure direct contact between the gel electrode and the scalp. To ensure the accuracy of the stimulation target point, each wearing was positioned and adjusted according to the international 10–20 system electrode placement method. The headset wearing process was consistent in both groups, followed by switching on the power supply of the tDCS stimulator and uniformly adjusting the constant current size to 1.8 mA. The stimulation time was set to 20 minutes in the intervention group and 30 seconds in the control group. The fade-in/fade-out of the current during the 30 seconds was designed to minimise skin sensations at the beginning and end of the stimulation so that the control group would produce a similar subjective sensation to that of the intervention group (14), to maintain the integrity of the blinded study.

The music module, which constitutes the main body of the headset, is used to play standardized musical materials. The music intervention materials used in this study were selected from the emotional music library developed by Chen et al. (31). This library comprises 200 instrumental works composed by 31 Western classical music masters, primarily piano pieces supplemented by instrumental duets and symphonic works. All participants listened to the same standardized playlist throughout the intervention to eliminate the influence of individual differences in musical preferences on the intervention’s effectiveness. The playlist consisted of 20 stress-relieving and sleep-inducing tracks, characterized by major keys, predominantly 3/4 and 4/4 time signatures, tempos within the andante range (60–80 beats per minute), and smooth, continuous melodies. Each track lasted approximately 2–3 minutes and was played on a loop. The music was streamed via a Bluetooth-connected smartphone, with the volume uniformly set to 40–50 dB to ensure participants could hear it clearly without feeling disturbed.

All sessions were conducted during the day (between 9:00 a.m. and 5:00 p.m.) to assess the cumulative effects of the intervention rather than the immediate hypnotic effects. Each session lasted 20 minutes, once a day, five days a week, for a total of four weeks. During the trial, participants in both groups were treated according to the established protocol and no other treatment was performed.

### 2.6 Outcome measurement

The primary outcome of this study was PSQI. The secondary outcomes included Self-rating Depression Scale (SDS), Self-rating Anxiety Scale (SAS), Perceived Stress Scale (PSS-14), and fNIRS data during VFT. On the day before treatment and the second day after four weeks of treatment, two trained and licensed physiotherapists evaluated the sleep quality, mental health, and brain function of patients in both groups. All results were averaged to ensure consistency and reliability of the assessments.

#### 2.6.1 PSQI

The scale contains 19 self-assessment items, which can be divided into 7 sub-dimensions: sleep quality, sleep latency, sleep duration, sleep efficiency, sleep disturbance, hypnotic drugs, and daytime dysfunction. To control for variables, subjects taking psychotropic medications were excluded from this study. Therefore, the hypnotic drug application dimension was not included in the analysis of results. The total score of the scale ranges from 0 to 21, with ≤5 indicating good sleep quality and >5 indicating a sleep disorder. Higher scores indicate poorer sleep quality (30). The Chinese version of the PSQI has been proven to have good reliability, validity, and internal consistency (Cronbach α = 0.84) (32).

#### 2.6.2 SDS

It was used to reflect the subjective experience of patients’ depression (33). According to the Chinese norm, the degree of depression was classified as “light”, “moderate”, and “severe” based on the score ranges of 53-62, 63-72, and ≥73. Higher scores represent more severe depression (34). Gabrys et al. demonstrated that the SDS has good reliability (Cronbach’s α = 0.88-0.93) in different populations (35).

#### 2.6.3 SAS

It was used to measure the subjective severity of the patient’s anxiety state (36). The Chinese standard classifies anxiety as “light”, “moderate”, and “severe” according to the score ranges of 50-59, 60-69, and ≥70, with higher scores indicating more severe anxiety (37). Previous studies have shown that the Chinese version of the SAS has satisfactory internal consistency (Cronbach α = 0.82) (38).

#### 2.6.4 PSS-14

The assessment form contains 14 entries on a 5-point scale. The higher the score, the higher the perceived stress level of the individual (39). Scores 14-28, 29-42, 43-56, and 57–70 represent perceived stress levels of “low”, “moderate”, “high” and “very high”, respectively. Huang et al.’s study on a Chinese community sample showed that the PSS-14 has high reliability (Cronbach’s α = 0.83) (40).

#### 2.6.5 fNIRS functional brain imaging

This study employed the VFT as the fNIRS measurement paradigm. This task specifically activates the prefrontal cortex, which is associated with executive function and emotional regulation, as well as temporal lobe regions involved in language processing and semantic retrieval. Previous studies have demonstrated its high sensitivity in detecting prefrontal dysfunction in ID patients (20, 21). A brain functional imaging system (Bairuixin Intelligent Technology Co., Ltd., model: C01A, sampling frequency 5 Hz) was used to record the activity of the cerebral cortex during the verbal fluency task in the form of 785 nm and 825 nm continuous waves. Brain region localisation was performed based on the International EEG 10/20 system. The fNIRS fibre-optic cap consisted of 15 light sources and 16 detectors, generating 48 channels covering bilateral frontal and temporal cortex areas of the brain. They were further divided into 9 regions of interest (ROI) based on coordinate information: bilateral superior frontal cortex (SFC), bilateral DLPFC, bilateral superior temporal cortex (STC), bilateral ventrolateral prefrontal cortex (VLPFC), and medial prefrontal cortex (mPFC) (**Figures 2B, C**).

The VFT concluded a 25-second pre-task rest period, a 60-second task period, and a 55-second post-task rest period. The 60-second task period consisted of three consecutive 20-second task blocks, each using a different cue character, with no inter-block rest intervals. During the task, patients were asked to form as many phrases as possible using 3 random Chinese characters, such as “笔” (pen), “天” (sky), and “白” (white) for 20 seconds at a time. During breaks before and after the task, patients were asked to verbally repeat “1, 2, 3, 4, 5” until the end of the task. Before starting, the researcher explained the test procedure to the patient to ensure that the patient was proficient. After the patient was fitted with a fibre-optic cap, channel-by-channel commissioning and calibration were performed to ensure high signal quality. fNIRS testing was performed in a quiet, dedicated consulting room, and the equipment was regularly checked and maintained by professional staff. Patients were instructed the day before the test to ensure adequate sleep and avoid stimulating foods such as tea, alcohol, coffee, and tobacco.

#### 2.6.6 fNIRS data processing and analysis

The fNIRS data were processed using the brain functional imaging system software (Bairuixin Intelligent Technology Co., Ltd.). Firstly, motion artefacts were identified and removed. A Butterworth low-pass filter (0.1 Hz) was selected to filter out high-frequency noise and slow-drifting interfering signals, and panning was performed based on the minimum value of the last 10 seconds of data of the baseline state. Finally, the average changes in Oxy-haemoglobin (HbO_2_) concentration were calculated for each channel. Examined and confirmed fNIRS data from each participant with at least 36 high signal quality channels were considered valid for inclusion in the statistical analyses. The Shapiro-Wilk test was used to confirm the normality of the data. Differences in baseline and task-state HbO_2_ were compared using paired-sample t-tests to assess brain activation during the task. Based on task-state HbO_2_, Pearson correlation analysis was used to calculate FC between channel pairs and different brain regions to construct brain functional networks. Multiple comparisons of HbO_2_ concentrations across 48 channels and FC strengths between brain regions and between channel pairs were corrected using the false discovery rate (FDR) method with the Benjamini-Hochberg procedure. Statistical significance was defined as FDR-corrected *p* < 0.05.

### 2.7 Safety Assessment

The safety of the participants during treatment and any possible adverse effects were monitored throughout the study. Patients were asked how they felt before and after each intervention and any discomfort was recorded in detail. Patient status was closely monitored during treatment and any abnormalities were dealt with immediately. After each session, the electrode contact areas on the scalp were visually inspected by the therapists to rule out any potential skin reactions such as micro-burns. Patients were also asked to report any adverse reactions directly related to tDCS and/or music listening, such as scalp itching, tingling, dizziness, tinnitus, and vomiting. The data collected on adverse reactions were pooled and analysed to evaluate the side effects and safety of the treatment.

### 2.8 Statistical analysis

Statistical analysis was performed using SPSS 27.0 software. Data normality was confirmed by the Shapiro-Wilk test. Continuous data were reported as mean (standard deviation) to demonstrate data differences. Baseline data were compared using independent samples t-test and chi-square test. For continuous data with normal or non-normal distributions, within-group comparisons were performed using the paired-samples t-test or Wilcoxon signed-rank test, respectively, and comparisons of between-group differences were performed using the independent-samples t-test or the Mann-Whitney U test, respectively. Effect sizes were quantified using Cohen’s *d* (for parametric tests) or *r* (for nonparametric tests). Pearson’s correlation was used to explore the relationship between changes in HbO_2_ and FC and improvement in PSQI score. *p* < 0.05 was used as the significance level.

## 3 Results

### 3.1 Demographic characteristics of participants

The flow chart of this study is shown in **Figure 1**. Eligibility was assessed for 102 participants and 76 patients were finally included. During the trial, 6 participants in each of the intervention and control groups were lost to visit or did not complete treatment (personal factors unrelated to the trial), and 32 patients in each of the two groups were finally included for outcome analysis. There were no statistically significant differences between the two groups in terms of age, gender, years of education, and disease duration (*p* > 0.05), as shown in **Table 1**. Consistent with the exclusion criteria, no enrolled patient reported use of psychotropic medications requiring a washout period longer than 2 weeks (e.g., fluoxetine, MAOIs, or long-acting injectable antipsychotics). During treatment, 4 participants (12.5%) in the intervention group and 3 participants (9.4%) in the control group reported mild and transient scalp itching or tingling at the electrode sites. These sensations were quickly relieved after the stimulation, without any special treatment and did not affect daily life. There was no statistically significant difference in the incidence of adverse reactions between the two groups (χ² = 0.160, *p* = 0.689). Throughout the study, no treatment-related serious adverse events (such as seizures or syncope) or pain were observed. No patients discontinued treatment or withdrew from the study due to adverse reactions. In addition, the assessment of the blinding effectiveness showed that the intervention group had a correct grouping guess rate of 46.9% (15/32), while the control group had a rate of 43.8% (14/32). There was no statistically significant difference between the two groups in terms of correct guessing rates (χ² = 0.063, *p* = 0.802), and neither rate was significantly higher than the 50% random probability level, indicating that the blinding procedure in this study was effective.

**Table 1:** Comparison of demographic and baseline data between two groups.

Characteristics	Intervention group (N = 32)	Control group (N = 32)	*t/χ^2^*	*p*
Age, mean (SD), year	42.1 (13.9)	39.9 (14.8)	0.609	0.545
Sex (male/female), n	11/21	13/19	0.267	0.606^a^
Education, mean (SD), year	13.4 (5.2)	13.1 (4.5)	0.230	0.819
Disease duration, mean (SD), month	15.0 (11.8)	13.8 (12.2)	0.428	0.670
Scale scores, mean (SD)				
PSQI	13.3 (2.9)	12.9 (2.8)	0.524	0.602
SDS	52.2 (12.7)	54.8 (14.1)	-0.791	0.432
SAS	47.9 (11.3)	50.3 (16.5)	-0.689	0.493
PSS-14	42.6 (7.1)	41.4 (7.5)	0.649	0.519

PSQI, Pittsburgh Sleep Quality Index; SDS, Self-rating Depression Scale; SAS, Self-rating Anxiety Scale; PSS-14, Perceived Stress Scale.

^a^by chi-square test; otherwise, by Independent Samples *t*-test.

### 3.2 Sleep quality

At baseline, there was no significant difference in the total PSQI score between the two groups (*p* > 0.05). After treatment, both groups showed a significant reduction in PSQI scores (*p* < 0.001), with the intervention group demonstrating a significantly greater reduction than the control group (mean difference: -2.57 points, 95% CI: -4.43 to -0.71, *p* = 0.001). Regarding the PSQI sub-dimensions, no significant differences were observed between the two groups at baseline (all *p* > 0.05). Analysis of the dimensions showed a significant decrease in sleep quality, sleep latency, sleep duration, sleep efficiency, sleep disturbance, and daytime dysfunction sub-scores in the intervention group (*p* < 0.05). In the control group, sleep quality, sleep duration, sleep efficiency, and daytime dysfunction sub-scores decreased significantly (*p* < 0.05). Among them, the intervention group was significantly better than the control group in improving sleep quality, sleep latency, sleep duration, and sleep efficiency (*p* < 0.05) (**Table 2**).

**Table 2:** Comparison of scale scores between two groups pre- and post-treatment (points).

Variable	Group	Baseline	4 weeks	Change mean (95% CI)	Cohen’s *d*/*r* (95% CI)	*t*/*Z*	*p*-value between groups
PSQI total score	Intervention	13.25 (2.91)	8.56 (3.65)	-4.69 (-6.04 to -3.33)^c^	-0.854 (-1.363 to -0.338)	-3.416	**0.001**
	Control	12.88 (2.81)	10.75 (3.27)	-2.13 (-2.83 to -1.42)^c^			
Sleep quality^d^	Intervention	2.41 (0.56)	1.03 (0.82)	-1.38 (-1.66 to -1.09)^c^	0.368 (0.134 to 0.563)	-2.942	**0.003**
	Control	2.47 (0.67)	1.66 (0.70)	-0.81 (-1.03 to -0.60)^c^			
Sleep latency^d^	Intervention	2.28 (0.52)	1.78 (0.71)	-0.50 (-0.76 to -0.24)^b^	0.379 (0.147 to 0.571)	-3.031	**0.002**
	Control	2.13 (0.61)	2.09 (0.69)	-0.03 (-0.18 to 0.11)			
Sleep duration^d^	Intervention	2.47 (0.57)	1.59 (0.91)	-0.88 (-1.18 to -0.58)^c^	0.253 (0.008 to 0.470)	-2.023	**0.043**
	Control	2.25 (0.57)	1.75 (0.72)	-0.50 (-0.68 to -0.32)^c^			
Sleep efficiency^d^	Intervention	2.41 (0.62)	1.47 (0.88)	-0.94 (-1.25 to -0.62)^c^	0.300 (0.058 to 0.508)	-2.397	**0.017**
	Control	2.25 (0.57)	1.78 (0.71)	-0.47 (-0.65 to -0.29)^c^			
Sleep disturbance^d^	Intervention	1.72 (0.96)	1.34 (0.87)	-0.38 (-0.65 to -0.10)^a^	0.236 (-0.010 to 0.456)	-1.891	0.059
	Control	1.72 (1.09)	1.69 (0.97)	-0.03 (-0.23 to 0.16)			
Daytime dysfunction^d^	Intervention	1.97 (0.82)	1.34 (0.83)	-0.63 (-0.94 to -0.31)^b^	0.218 (-0.029 to 0.440)	-1.743	0.081
	Control	2.06 (0.67)	1.78 (0.87)	-0.28 (-0.49 to -0.07)^a^			
SDS	Intervention	52.16 (12.73)	44.38 (10.81)	-7.78 (-10.54 to -5.02)^c^	-0.569 (-1.066 to -0.066)	-2.274	**0.026**
	Control	54.81 (14.10)	51.50 (13.48)	-3.31 (-6.22 to -0.41)^a^			
SAS	Intervention	47.88 (11.32)	39.25 (10.76)	-8.63 (-11.28 to -5.97)^c^	-0.586 (-1.084 to -0.083)	-2.343	**0.022**
	Control	50.31 (16.50)	45.94 (13.47)	-4.38 (-6.96 to -1.79)^b^			
PSS-14	Intervention	42.56 (7.12)	38.19 (8.60)	-4.38 (-7.11 to -1.64)^b^	-0.373 (-0.866 to 0.122)	-1.494	0.140
	Control	41.38 (7.53)	39.44 (5.67)	-1.94 (-3.84 to -0.04)^a^			

^a^ *p* < 0.05, ^b^*p* < 0.01, ^c^*p* < 0.001 compared with baseline by paired sample *t*-test or ^d^Wilcoxon signed-rank test.

Mean (SD).

Bolded values indicate statistically significant differences.

### 3.3 Mental health

Before treatment, there was no significant difference between the SDS, SAS, and PSS-14 scores of the two groups (*p* > 0.05). After treatment, the SDS and SAS scores of both groups decreased significantly (*p* < 0.05), and the intervention group improved better than the control group (*p* < 0.05). After treatment, PSS-14 scores decreased significantly in both groups (*p* < 0.05). However, there was no significant difference between the two groups (*p* > 0.05) (**Table 2**).

### 3.4 Brain activation and functional connectivity changes

**Figure 3:**
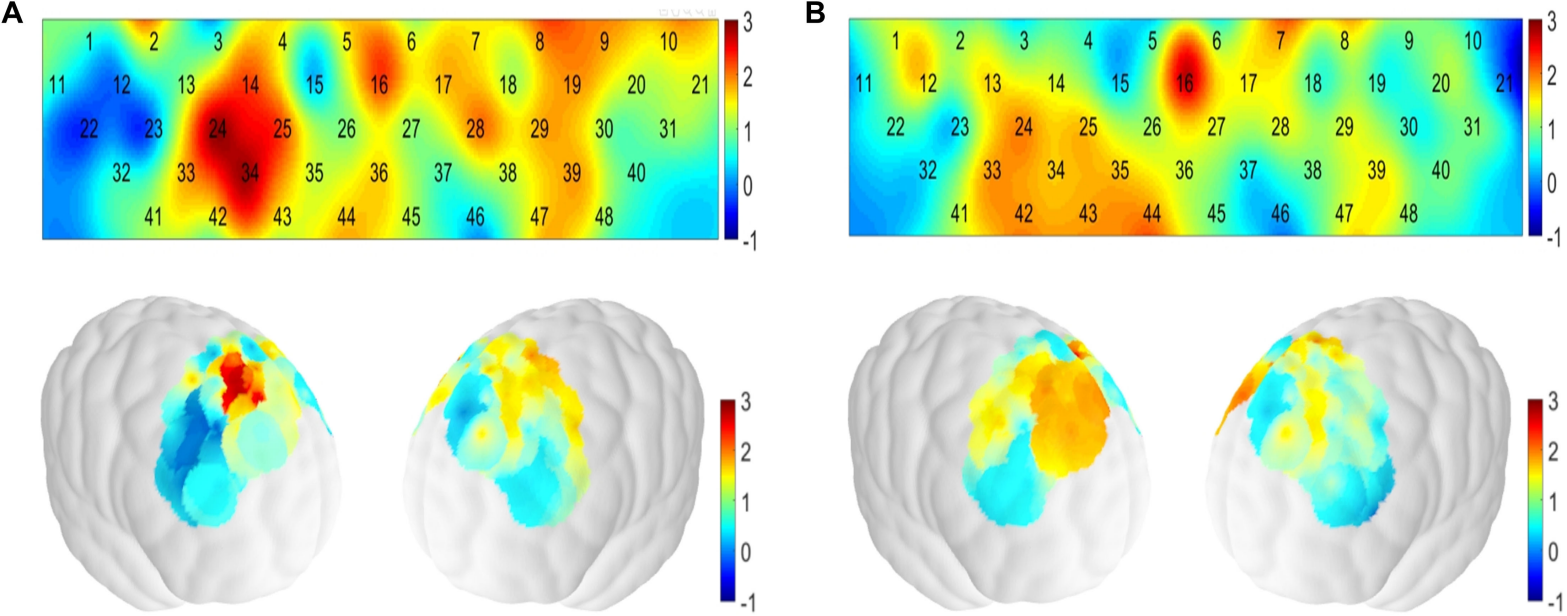
Differences in channel activation during the verbal fluency task before and after treatment in both groups. **(A)** The intervention group. **(B)** The control group. The legend shows t-values from paired-sample t-tests.

Following preprocessing and channel quality checks, according to the predefined inclusion criteria (each participant required at least 36 channels with high signal quality both before and after intervention), fNIRS data from 27 participants in the intervention group and 29 in the control group were ultimately included in the outcome analysis. The remaining participants were excluded due to poor signal quality. The difference in activation of 48 channels during the performance of the VFT before and after treatment between the two groups is shown in (**Figure 3**). In the intervention group, significant post-treatment activation was observed in 6 channels in the prefrontal regions, including the lDLPFC, rDLPFC, mPFC and rVLPFC (**Figure 3A**). In the control group, significant activation was observed in 3 channels, mainly in the lDLPFC and mPFC (**Figure 3B**). The changes in HbO_2_ concentration in the 9 brain regions before and after treatment are shown in **Table 3**. The HbO_2_ concentrations of rSFC, lDLPFC, rVLPFC, and mPFC in the intervention group, and rVLPFC in the control group increased significantly after treatment. However, there was no significant difference between the two groups (*p* > 0.05).

**Table 3:** Changes in mean HbO_2_ concentration in different brain regions pre-and post-treatment in both groups (mmol/L*mm).

Brain region	Group	Baseline	4 weeks	Change mean (95% CI)	Within-group *p*	Between-group *t*	Between-group *p*
rSFC	Intervention	0.033 (0.109)	0.059 (0.091)	0.026 (-0.021 to 0.073)	0.261	-0.225	0.823
	Control	0.003 (0.094)	0.037 (0.097)	0.034 (-0.017 to 0.084)	0.184		
lSFC	Intervention	-0.018 (0.144)	0.041 (0.087)	0.059 (0.005 to 0.114)	**0.033**	0.789	0.433
	Control	-0.014 (0.160)	0.014 (0.079)	0.028 (-0.034 to 0.089)	0.367		
rDLPFC	Intervention	0.001 (0.169)	0.049 (0.073)	0.048 (-0.018 to 0.114)	0.150	-0.016	0.987
	Control	-0.017 (0.158)	0.032 (0.117)	0.048 (-0.026 to 0.123)	0.194		
lDLPFC	Intervention	-0.024 (0.132)	0.041 (0.075)	0.066 (0.018 to 0.114)	**0.009**	0.479	0.634
	Control	-0.026 (0.158)	0.020 (0.088)	0.047 (-0.019 to 0.112)	0.155		
rSTC	Intervention	0.040 (0.100)	0.040 (0.111)	0.000 (-0.058 to 0.059)	0.990	-0.324	0.747
	Control	0.037 (0.097)	0.049 (0.092)	0.012 (-0.031 to 0.055)	0.580		
lSTC	Intervention	-0.018 (0.178)	0.014 (0.092)	0.032 (-0.029 to 0.093)	0.293	0.196	0.845
	Control	-0.004 (0.173)	0.019 (0.085)	0.023 (-0.047 to 0.093)	0.511		
rVLPFC	Intervention	-0.006 (0.136)	0.057 (0.084)	0.063 (0.008 to 0.118)	**0.026**	-0.045	0.964
	Control	-0.015 (0.135)	0.050 (0.093)	0.065 (0.003 to 0.127)	**0.040**		
lVLPFC	Intervention	-0.011 (0.168)	0.036 (0.085)	0.047 (-0.009 to 0.103)	0.095	0.271	0.787
	Control	0.009 (0.161)	0.044 (0.101)	0.035 (-0.033 to 0.104)	0.300		
mPFC	Intervention	-0.018 (0.111)	0.057 (0.063)	0.075 (0.027 to 0.124)	**0.004**	0.671	0.505
	Control	-0.014 (0.147)	0.033 (0.095)	0.047 (-0.021 to 0.116)	0.169		

Mean (SD).

Bolded values indicate statistically significant differences.

**Figure 4:**
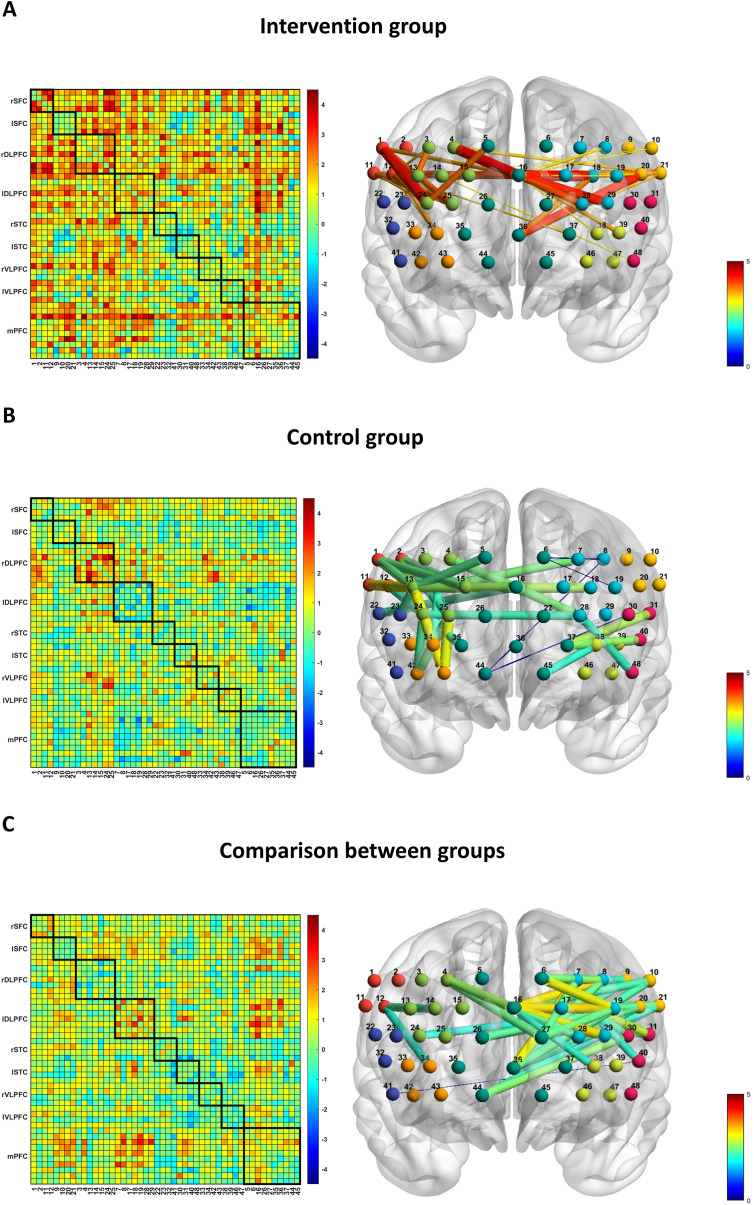
Differences in functional connectivity strength during the task before and after treatment in the intervention **(A)** and control **(B)** groups. The legend shows t-values from paired-sample t-tests. **(C)** Differences in changes in functional connectivity strength during the task before and after treatment between the two groups. The legend shows t-values from independent samples t-tests. The 3D brain images were visualized with the BrainNet Viewer [(58), http://www.nitrc.org/projects/bnv/].

Functional connectivity analysis showed that the FC strength between 36 channel pairs in the intervention group was significantly higher after treatment than before (all *t* > 3.073, all *p* < 0.01) (**Figure 4A**). Meanwhile, the FC of rSFC-mPFC, lSFC-rDLPFC, lSFC-lDLPFC, lSFC-mPFC, and lSTC-mPFC was significantly enhanced (**Table 4**). The FC intensity of the 25 channel pairs in the control group was significantly higher after treatment than before (all *t* > 2.058, all *p* < 0.05) (**Figure 4B**). Meanwhile, the FC between rSFC and rDLPFC was significantly enhanced (**Table 4**). Comparison between the groups showed that the FC enhancement of the lSFC-lDLPFC and the lSFC-mPFC was significantly better than that of the control group after treatment in the intervention group (**Table 4**). In addition, we examined the 39 channel pairs in which there was a significant difference in FC intensity changes between the two groups (**Figure 4C**) and found that channel 16 appeared most frequently (10 times, belonging to the mPFC), followed by channel 36 (7 times, belonging to the mPFC) and channel 18 (7 times, belonging to the lDLPFC), with the specific values shown in **Supplementary Table 1**.

**Table 4:** Brain regions with significant differences in functional connectivity strength pre-and post-treatment in both groups.

Brain region	Group	Baseline	4 weeks	Change mean (95% CI)	Cohen’s *d* (95% CI)	*t*	*p*
Intervention							
rSFC-mPFC		0.379 (0.343)	0.638 (0.338)	0.259 (0.079 to 0.439)	-0.568 (-0.971 to -0.156)	2.952	**0.007**
lSFC-rDLPFC		0.344 (0.321)	0.536 (0.338)	0.192 (0.008 to 0.376)	-0.412 (-0.802 to -0.015)	2.142	**0.042**
lSFC-lDLPFC		0.591 (0.364)	0.786 (0.253)	0.195 (0.047 to 0.343)	-0.521 (-0.920 to -0.114)	2.709	**0.012**
lSFC-mPFC		0.371 (0.362)	0.660(0.308)	0.289 (0.128 to 0.450)	-0.711 (-1.129 to -0.282)	3.694	**0.001**
lSTC-mPFC		0.408 (0.375)	0.589 (0.325)	0.181 (0.010 to 0.353)	-0.419 (-0.809 to -0.021)	2.177	**0.039**
Control							
rSFC-rDLPFC		0.601 (0.367)	0.803 (0.269)	0.202 (0.015 to 0.389)	-0.411 (-0.029 to -0.788)	2.216	**0.035**
Intergroup							
lSFC-lDLPFC	Intervention			0.195 (0.047 to 0.343)	0.570 (0.033 to 1.103)	2.133	**0.037**
	Control			-0.029 (-0.185 to 0.127)			
lSFC-mPFC	Intervention			0.289 (0.128 to 0.450)	0.574 (0.037 to 1.107)	2.148	**0.036**
	Control			0.032 (-0.151 to 0.216)			

Mean (SD).

Bolded values indicate statistically significant differences.

### 3.5 Correlation of functional brain activity with improved sleep quality

**Figure 5:**
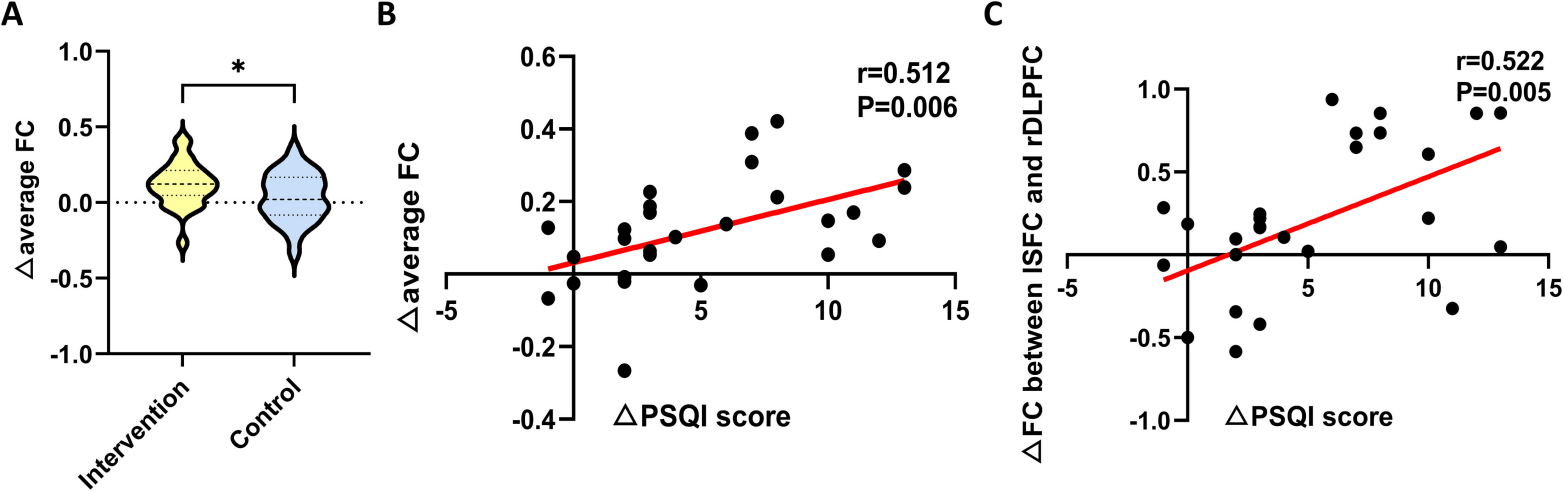
**(A)** Comparison of average functional connectivity changes over 48 channels after treatment between the two groups. Correlation analysis of changes in the average **(B)** and the lSFC-rDLPFC **(C)** functional connectivity with changes in PSQI score in the intervention group, respectively. ΔPSQI, changes in PSQI score before and after treatment; ΔFC, changes in functional connectivity before and after treatment. *p < 0.05.

After treatment, the mean FC of the 48 channels significantly increased in the intervention group (*t* = 4.266, *p* < 0.001) and improved better than in the control group (0.120 ± 0.046 vs 0.035 ± 0.162, *t* = 2.060, *p* = 0.044). In addition, correlation analyses showed a positive correlation between the improvement in PSQI score and the enhancement of mean FC (r = 0.512, *p* = 0.006) and FC of lSFC-rDLPFC (r = 0.522, *p* = 0.005) in the intervention group (**Figure 5**).

## 4 Discussion

To the best of our knowledge, this is the first time that tDCS has been used in combination with music listening in the form of musical electrical stimulation headphones in ID patients. It is also the first time that neuroplasticity changes in haemodynamic responses during VFT in ID patients after combined treatment are reported using fNIRS. As we hypothesized, the combination of bifrontal-tDCS with music listening helps to improve the subjective sleep quality of ID patients, reduces adverse moods such as depression and anxiety, and enhances prefrontal neural network functional connectivity, with overall therapeutic efficacy superior to music listening alone. The stimulation effect of tDCS depends on the cortical target area and stimulation parameters, including stimulation current density, duration, and number of repetitions (41). The neural bioelectrical activity of the prefrontal regions of the brain, especially the DLPFC, plays an important role in cognitive function, sleep, and emotion regulation (42). Therefore, in this study, the bilateral DLPFC was chosen as the target of repetitive stimulation in combination with music stimulation.

The results of this study showed that the total PSQI scores of both groups were lower than before treatment, and the intervention group improved better than the control group. Meanwhile, the intervention group was superior to the control group in improving sleep quality, sleep latency, sleep duration, and sleep efficiency. This suggests that combining tDCS with music listening improves subjective sleep quality in ID patients and benefits them in almost all dimensions. Similar to our findings, a study used polysomnography (PSG) to monitor patients with major depression and insomnia treated with 4 weeks of bifrontal tDCS (F3: anodal, F4: cathodal, 2 mA, 30 min). The results showed a significant improvement in the total PSQI score and all sub-divisions except “sleep duration and sleep efficiency” in the active stimulation group compared to the sham stimulation group, and the PSG showed an increase in total sleep duration and sleep efficiency (15).

Studies by Plante et al. (43) and Allen et al. (44) found that insomnia patients had lower levels of γ⁃aminobutyric acid and serotonin and higher levels of glutamate, which may be associated with the generation and development of adverse moods such as depression and anxiety. The present study showed that depression and anxiety improved in both groups after treatment, and the intervention group had better efficacy than the control group but did not show a significant advantage for stress perception. It has been shown that anodal stimulation enhances γ⁃aminobutyric acid release through subthreshold depolarization and also modulates the dopamine system, enhancing and inhibiting serotonin and acetylcholine transmission, respectively, whilst cathodal stimulation inhibits glutamate transmission, thus modulating the release of neurotransmitters and the balance of excitability of the neural network (45). Music listening has synergistic effects on mood, arousal states, and neural network modulation (46). Mansouri et al. found interactive effects between music and tDCS in the prefrontal cortex in modulating mood states and the ability to improve learning and executive functioning based on altered arousal-emotional responses to decision outcomes (47).

In this study, we found that the activation of mPFC, lDLPFC, lSFC, and rVLPFC was significantly increased after treatment in the intervention group, but there was no significant difference compared with the control group. This suggests that combined therapy can enhance local neural activity in the prefrontal cortex, but has not yet demonstrated superiority over music listening alone in this regard, indicating that local activation may not be the primary mechanism underlying the benefits of combined therapy. Zhou et al. found that cortical activation of bilateral mPFC and DLPFC was significantly reduced in ID patients, and there was a significant negative correlation between the PSQI score and the mean HbO_2_ of the mPFC (25). Reduced activation in the bilateral frontal and temporal cortex, bilateral parahippocampal gyrus, and other areas may be neurobiological markers of emotional and cognitive dysfunction in patients with ID (48). Giovannella et al. found that tDCS increased cerebral blood flow and HbO_2_ content, decreased deoxyhaemoglobin levels, and improved local blood flow regulation in the brain (49). In our study, we also found that the combined therapy helps enhance excitability and hemodynamic responses in the prefrontal cortex, thereby establishing a neural basis for improved sleep.

It is worth noting that, although there were no significant differences in HbO_2_ activation between the groups, the intervention group demonstrated significantly greater improvements than the control group at the functional connectivity level. The core pathological change underlying insomnia is not merely insufficient activation in a single brain region, but rather disrupted connectivity within the sleep regulation network centred on the prefrontal cortex (50). As an indicator of the temporal correlations between neural activity in different brain regions, FC can reveal the collaborative mechanisms of the brain as a dynamically interconnected network. By FC analysis, we observed a significant increase in mean FC and FC between bilateral SFC, mPFC, and bilateral DLPFC in the intervention group. Meanwhile, the FC enhancement of the lSFC-lDLPFC and the lSFC-mPFC in the intervention group was significantly better than that in the control group. The finding that cortical activation showed no significant difference while FC was significantly enhanced suggests that the unique effect of the combined therapy may lie in optimizing the coordination efficiency of networks between brain regions, rather than simply increasing the activity of local brain regions. In other words, the combined therapy may restore the connectivity of impaired cortical and subcortical neural networks by improving functional coordination among the SFC, DLPFC and mPFC. In addition, the improvement in sleep quality of patients in the intervention group was positively correlated with the increase in mean FC and FC between left SFC and right DLPFC. The SFC plays a crucial role in motor control, cognitive function, and emotional regulation. Studies have shown that prefrontal cortex activity directly regulates homeostatic sleep pressure (51), and that SFC activation levels during the VFT are positively correlated with sleep efficiency in patients with insomnia (52). Based on this evidence, changes in functional connectivity within the prefrontal executive network during wakefulness may indirectly contribute to the remodelling of sleep regulatory mechanisms, potentially by modulating synaptic efficacy in prefrontal cortex neurons. Previous studies have demonstrated that decreased rDLPFC function is associated with reduced secretion of sleep-regulating hormones, ultimately leading to a hyperarousal state in ID patients (26, 53). On this basis, we speculate that restoring the abnormal functional connectivity between the rDLPFC and lSFC may reduce arousal levels, improve sleep quality, and enhance daytime function by enhancing prefrontal synaptic efficacy and normalizing the secretion of sleep-regulating hormones.

From a mechanistic perspective, the combined effects of tDCS and music listening are reflected in this network-level integration. Specifically, tDCS provides “top-down” cortical regulation by directly stimulating the bilateral DLPFC, while music listening activates the limbic system via auditory pathways, thereby achieving “bottom-up” regulation of emotional and cognitive responses (46). Anodal tDCS increases cortical excitability, making individuals more sensitive to musical perception and emotional resonance (54); musical stimulation further drives plasticity changes in cortical and subcortical structures, enhancing the neuromodulatory effects of tDCS (55, 56). The combination of the two promotes functional coupling within the prefrontal cortex and between cortical and subcortical neural circuits, ultimately contributing to improved sleep quality. At the same time, the therapeutic form of musical electrical stimulation headphones is more easily accepted by the general public, which is conducive to improving patient compliance. In addition, there were no significant side effects in the trial except for itching and mild tingling sensations reported by patients, which is a high level of safety (57). This may be because the trial was operated in the hospital by professionals who strictly followed the guidelines for the use of the equipment and explicitly excluded patients who were not suitable for tDCS treatment.

This study has some limitations due to single-centre recruitment, small sample size, lack of long-term follow-up and objective sleep quality assessment (e.g. PSG/EEG). In addition, the single-block VFT paradigm may have limited signal-to-noise ratio. Further research and studies are needed to investigate the duration of maintenance of efficacy and long-term effects. Nonetheless, this study is the first to assess and validate the effectiveness of tDCS combined with music listening to improve insomnia, providing a relatively safe and effective neuromodulation method for insomnia treatment, and providing new empirical support for insomnia-related neuroimaging. It may ultimately contribute to the clinical diagnosis, treatment, and home management of insomnia.

## 5 Conclusion

Our study suggests that combined bifrontal-tDCS with music listening is more helpful in improving sleep quality and alleviating depressive and anxiety symptoms compared with music listening alone. Preliminary evidence suggests that musical electrical stimulation headphones may be a safe, effective, and convenient new treatment strategy for insomnia disorder, potentially through enhancing prefrontal functional connectivity, particularly between the left SFC, DLPFC, and mPFC.

## Data Availability

The raw data supporting the conclusions of this article will be made available by the authors, without undue reservation.
