# Supplementary material for: Effects of bifrontal-transcranial direct current stimulation combined with music listening on sleep quality, cortical activation and functional connectivity in patients with insomnia: a randomised controlled trial by fNIRS
Source: Front Psychiatry. 2026 Apr 29;17:1763543. doi: 10.3389/fpsyt.2026.1763543 (PMC13167946; doi:10.3389/fpsyt.2026.1763543)
Supplement: Supplementary file 1 [file Table1.docx]

**Supplementary Table 1** Channel pairs with significantly different changes in functional connectivity strength before and after treatment in both groups. Mean (SD)

| Channel number | Brain region | Functional connectivity changes | | *t* | *p* (FDR corrected) |
| --- | --- | --- | --- | --- | --- |
|  |  | Intervention | Control |  |  |
| 4-16 | rDLPFC-mPFC | 0.361±0.419 | -0.010±0.587 | 2.657 | 0.012 |
| 4-27 | rDLPFC-mPFC | 0.209±0.575 | -0.117±0.430 | 2.371 | 0.012 |
| 6-8 | mPFC-lDLPFC | 0.147±0.521 | -0.230±0.553 | 2.573 | 0.012 |
| 6-9 | mPFC-lSFC | 0.140±0.440 | -0.138±0.534 | 2.077 | 0.013 |
| 6-19 | mPFC-lDLPFC | 0.189±0.522 | -0.255±0.556 | 3.018 | 0.008 |
| 7-27 | lDLPFC-mPFC | 0.164±0.489 | -0.167±0.554 | 2.325 | 0.012 |
| 7-36 | lDLPFC-mPFC | 0.229±0.386 | -0.127±0.446 | 3.128 | 0.007 |
| 8-16 | lDLPFC-mPFC | 0.296±0.451 | -0.111±0.466 | 3.259 | 0.007 |
| 8-27 | lDLPFC-mPFC | 0.219±0.481 | -0.084±0.498 | 2.276 | 0.012 |
| 8-36 | lDLPFC-mPFC | 0.235±0.420 | -0.065±0.419 | 2.631 | 0.012 |
| 9-27 | lSFC-mPFC | 0.169±0.447 | -0.088±0.465 | 2.069 | 0.013 |
| 10-16 | lSFC-mPFC | 0.273±0.446 | -0.040±0.569 | 2.243 | 0.012 |
| 10-36 | lSFC-mPFC | 0.178±0.369 | -0.094±0.500 | 2.257 | 0.012 |
| 12-14 | rSFC-rDLPFC | 0.340±0.565 | 0.004±0.564 | 2.192 | 0.012 |
| 12-34 | rSFC-rVLPFC | 0.342±0.509 | 0.063±0.511 | 2.012 | 0.013 |
| 16-18 | mPFC-lDLPFC | 0.332±0.385 | -0.022±0.571 | 2.654 | 0.012 |
| 16-19 | mPFC-lDLPFC | 0.290±0.464 | -0.169±0.532 | 3.370 | 0.007 |
| 16-21 | mPFC-lSFC | 0.306±0.435 | -0.028±0.560 | 2.429 | 0.012 |
| 16-28 | mPFC-lDLPFC | 0.360±0.465 | 0.062±0.496 | 2.270 | 0.012 |
| 16-29 | mPFC-lDLPFC | 0.373±0.449 | -0.053±0.504 | 3.270 | 0.007 |
| 16-31 | mPFC-lSTC | 0.219±0.403 | -0.089±0.499 | 2.475 | 0.012 |
| 16-38 | mPFC-lVLPFC | 0.291±0.467 | 0.011±0.540 | 2.030 | 0.013 |
| 18-20 | lDLPFC-lSFC | 0.311±0.495 | -0.001±0.495 | 2.316 | 0.012 |
| 18-27 | lDLPFC-mPFC | 0.243±0.485 | -0.082±0.517 | 2.372 | 0.012 |
| 18-30 | lDLPFC-lSTC | 0.166±0.570 | -0.157±0.490 | 2.234 | 0.012 |
| 18-31 | lDLPFC-lSTC | 0.272±0.479 | -0.098±0.474 | 2.857 | 0.011 |
| 18-36 | lDLPFC-mPFC | 0.336±0.454 | 0.063±0.524 | 2.037 | 0.013 |
| 18-40 | lDLPFC-lSTC | 0.203±0.557 | -0.084±0.473 | 2.047 | 0.013 |
| 19-26 | lDLPFC-mPFC | 0.200±0.511 | -0.117±0.507 | 2.285 | 0.012 |
| 19-27 | lDLPFC-mPFC | 0.226±0.491 | -0.107±0.533 | 2.382 | 0.012 |
| 19-36 | lDLPFC-mPFC | 0.234±0.446 | -0.061±0.461 | 2.381 | 0.012 |
| 20-26 | lSFC-mPFC | 0.197±0.441 | -0.110±0.502 | 2.386 | 0.012 |
| 20-27 | lSFC-mPFC | 0.235±0.392 | -0.070±0.547 | 2.343 | 0.012 |
| 20-36 | lSFC-mPFC | 0.284±0.346 | 0.014±0.525 | 2.214 | 0.012 |
| 21-24 | lSFC-rDLPFC | 0.236±0.412 | -0.012±0.486 | 2.019 | 0.013 |
| 21-36 | lSFC-mPFC | 0.241±0.354 | -0.023±0.529 | 2.134 | 0.013 |
| 21-44 | lSFC-mPFC | 0.227±0.437 | -0.021±0.442 | 2.066 | 0.013 |
| 30-44 | lSTC-mPFC | 0.123±0.419 | -0.176±0.448 | 2.532 | 0.012 |
| 40-41 | lSTC-rSTC | 0.136±0.406 | 0.097±0.422 | 2.069 | 0.013 |
